# De novo mosaic *MECP2* mutation in a female with Rett syndrome

**DOI:** 10.1002/ccr3.1985

**Published:** 2019-01-15

**Authors:** Angelos Alexandrou, Ioannis Papaevripidou, Ioanna Maria Alexandrou, Athina Theodosiou, Paola Evangelidou, Ludmila Kousoulidou, George Tanteles, Violetta Christophidou‐Anastasiadou, Carolina Sismani

**Affiliations:** ^1^ Department of Cytogenetics and Genomics The Cyprus Institute of Neurology and Genetics Nicosia Cyprus; ^2^ Department of Clinical Genetics The Cyprus Institute of Neurology and Genetics Nicosia Cyprus; ^3^ Archbishop Makarios III Medical Centre Nicosia Cyprus; ^4^ The Cyprus School of Molecular Medicine Nicosia Cyprus

**Keywords:** *MECP2* mutation, next‐generation sequencing, Rett syndrome, somatic mosaicism

## Abstract

We describe a female with Rett syndrome carrying a rare de novo mosaic nonsense mutation on *MECP2* gene, with random X‐chromosome inactivation. Rett syndrome severity in females depends on mosaicism level and tissue specificity, X‐chromosome inactivation, epigenetics and environment. Rett syndrome should be considered in both males and females.

## 1 INTRODUCTION

This study is the first report of a female patient carrying a rare *MECP2* point mutation in a mosaic form with typical RS phenotype. Copy number and methylation analysis excluded Prader‐Willi and Angelman syndromes. Sanger sequencing of *MECP2* gene revealed a de novo mosaic C>T nonsense mutation at position 139 of exon 3. In order to estimate the level of mosaicism, Next‐Generation Sequencing was performed, showing approximately 25% abnormal cells. X‐chromosome inactivation analysis of the patient showed a random pattern.

This is the first known female mosaic *MECP2* mutation carrier, with a classical RS phenotype. The specific mutation in its full non‐mosaic form was previously detected in only one RS patient.

Detailed investigation of rare mosaic patients is of significant value for the understanding of molecular mechanisms involved in tissue‐specific gene expression and development of RS clinical features.

Rett syndrome (RTT) was first described by Andreas Rett in 1966 as a progressive neurodevelopmental disorder.^1^ It currently accounts for a large portion of intellectual disability (ID) in females, with its prevalence estimated at 1:10 000 live female births.^2^ The main clinical features of RS include developmental regression affecting motor and language skills, stereotypical hand movements, microcephaly, and ataxia. The criteria necessary for RS diagnosis are normal prenatal and perinatal period; normal psychomotor development through the first 6 months of life; normal head circumference at birth, with subsequent deceleration of head growth; loss of purposeful hand skills; severely impaired expressive and receptive language; apparent severe intellectual disability; and gait apraxia and truncal apraxia/ataxia.^3^ The phenotypic variation observed in RS patients over the years allowed for a definition of “classical” and “atypical” forms, with their diagnostic boundaries more clearly set in 2010.^4^ A challenge for RS clinical diagnosis is the overlap of its phenotype with other known syndromes such as Angelman (AS; MIM # 105830), Pitt‐Hopkins (PTHS; MIM #610954), and Mowat‐Wilson (MWS; MIM # 235730).

Rett syndrome is an X‐linked dominant disorder, with the vast majority of cases being caused by mutations or deletions in the *MECP2* gene located on Xq28, coding for the methyl‐CPG‐binding protein 2.^5^ However, it has recently been shown that approximately 5% of patients with RS features may carry mutations within other genes such as cyclin‐dependent kinase‐like 5 (*CDLK5*) and forkhead box G1 (*FOXG1*), as well as other genes related to epilepsy or intellectual disability.^6^, ^7^, ^8^, ^9^

*MECP2* mutations can also be found in males, although at a lower frequency, since most full *MECP2* mutations and deletions are lethal at the foetal stage. Other types of full mutations within *MECP2* gene in males can result in a variety of phenotypes from mild ID to severe neonatal encephalopathy.^10^ In a more recent report, a male with classic RS was described to carry a 5 bp duplication in the open reading frame of exonh1 of *MECP2*, thus highlighting the importance of considering RS in males, as well as in females.^11^ In case of somatic *MECP2* mosaicism, male patients can survive to birth and exhibit clinical features similar to the female full mutation^12^ or an atypical RS phenotype.^13^, ^14^ The frequency of *MECP2* mosaicism is currently unknown, with only male mosaic patient being reported so far and to our knowledge, no reports have been published to date describing *MECP2* mutation mosaicism in females.

In this study, we present a unique case of a rare mosaic *MECP2* point mutation found in a female patient with typical Rett syndrome. This new finding will add to the understanding of pathogenic mechanisms leading to phenotype manifestation in somatic mosaicism and may assist in the predictions concerning RS severity.

## 2 MATERIALS AND METHODS

### 2.1 Patients and samples

The patient was a five‐year‐old girl at the time of referral, with a phenotype highly indicative of typical Rett syndrome, including autistic features, psychomotor delay, severe intellectual disability, normal MRI, speech regression, and stereotypic hand movements. The patient's biological parents are unaffected. The patient was referred for investigation of Prader Willi‐Angelman (PWA) syndromes as part of consented diagnostic services.

DNA samples of the patient and both biological parents were obtained by isolation from peripheral blood using the QIAamp DNA Midi kit (Qiagen, Hidden, Germany) according to the supplier's protocol.

### 2.2 Genetic testing

Array comparative genomic hybridization (array‐CGH) was conducted using the SurePrint ISCA array (Agilent‐version 2.0) containing 60 000 oligos in an 8 × 60k format following the manufacturer recommendations (Agilent Technologies Inc., Santa Clara, CA, USA).

Methylationspecific multiplex ligation‐dependant probe amplification (MS‐MLPA) analysis for PWA was performed using MS‐MLPA Kit Prader Willi/Angelman (MRC Holland, Cat # ME028) according to the manufacturer's instructions.

Mutation analysis of all four coding exons and intron/exon boundaries of the *MECP2* gene were carried out using PCR amplification and direct sequencing, as described in Appendix S1.

Parental testing was performed using primer sequences for Exon 3 and confirmed by restriction enzyme analysis using the *Bbv1* enzyme (New England BioLabs Inc., Ipswich, MA, USA).

Next‐Generation Sequencing (NGS) was performed targeting, among others, all coding regions of *MECP2* and *FOXG1*, using the Nextera XT DNA Library preparation kit (Illumina, San Diego, CA, USA) with the obtained PCR amplicons on NextSeq 500 system according to the manufacturer's protocol (Illumina Inc).

The X‐chromosome inactivation (XCI) pattern was determined by methylation analysis for the region flanking the polymorphic CAG repeat in exon 1 of the *AR* gene.^15^

### 2.3 NGS Bioinformatics analysis

The NGS bioinformatics analysis was carried out using our in‐house pipeline https://training.vi-seem.eu/images/trainingMaterial/LifeSciences/NGS_analysis_pipeline_Theodosiou_CYI.pdf that follows GATK best practice recommendations (https://software.broadinstitute.org/gatk/). More details are given in Appendix S1.

## 3 RESULTS

**Figure 1:**
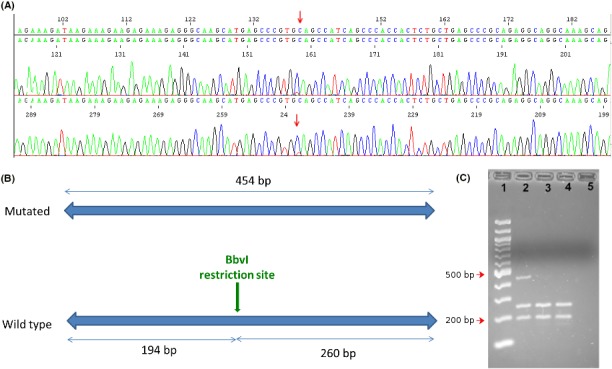
A, Sanger sequencing results revealing a C→T mutation at position 139 in exon 3 in the patient. B, Schematic illustration of *Bbv1* restriction site presence on the wild type sequence and absence on the mutated sequence. Wild type sequence restriction is expected to result in a 194 and 260 bp fragment, while the fragments carrying the mutation remain at 454 bp. C, Electrophoresis after restriction enzyme analysis with *Bbv1*. Lanes: 1—100 bp ladder; 2‐patient; 3‐mother; 4‐father; 5‐blank

**Figure 2:**
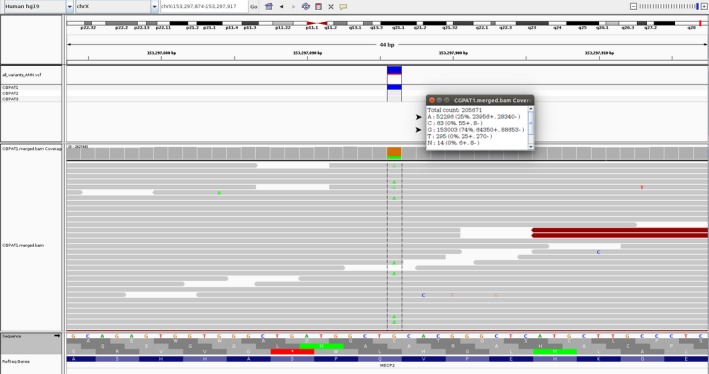
IGV illustration of the NGS results on the Forward strand of Human genome, with mosaicism percentage estimation (indicated by red arrows)

Array‐CGH analysis of the patient, as well as MS‐MLPA for PWA resulted in no abnormal findings (data not shown). The phenotype of the patient was highly indicative of RS; therefore, targeted Sanger sequencing of *MECP2* gene was performed and subsequently revealed a de novo mosaic C>T mutation at position 139 in exon 3, transcript NM_004992 (Figure 1). The aberration is a nonsense mutation predicted to be p.Gln47X at the protein level. Restriction enzyme analysis gave normal results for both parents and also confirmed the mutation in the patient (Figure 1). The level of mosaicism was estimated with NGS to be approximately 25% (Figure 2).

X‐chromosome inactivation (XCI) analysis of the patient showed a random XCI pattern.

## 4 DISCUSSION

In this case report, we describe a female patient with a phenotype highly indicative of RS, carrying a rare mosaic *MECP2* mutation. This c.139C=/>T de novo nonsense mutation on exon 3 creates a premature stop codon resulting in a truncated protein. The mutation is rare and has only been reported once in the literature^16^ in a large study investigating RS genotype‐phenotype correlation. The presence of this mutation in its full non‐mosaic form was detected in a female patient with classical RS phenotype, and further studies have shown an 83% XCI ratio.^16^ Its localization within the coding region of the RS main causative gene in combination with its predicted truncating effect suggest that it is most probably causative of the phenotype described in our patient.

To the best of our knowledge, female *MECP2* mosaic mutations have not been described in the literature. Male individuals carrying causative *MECP2* mutations in a mosaic form have typical or mild/atypical RS, which is considerably milder than male carriers of the corresponding full mutations, who rarely survive to birth.^12^, ^14^ Extrapolating on female mosaics, one would theoretically expect a mild RS or a status of almost asymptomatic carrier. In our case, despite the relatively low level of mosaicism (~25%), the patient is exhibiting the full spectrum of Rett syndrome phenotype, including some key clinical features such as autistic traits, psychomotor delay, speech regression, and stereotypic hand movements.

This apparently unexpected severity may be partially explained by high penetrance of the new mutation due to its truncating nature. Truncating mutations within *MECP2* have been associated with a more severe RS manifestation, as compared to missense mutations which may cause a milder or atypical RS.^17^ On the same level, the patient's genetic background may also influence the severity of some symptoms attributed to RS.

In contrast to male mosaic *MECP2* mutation carriers, female mosaic phenotype is significantly influenced by X‐chromosome inactivation (XCI) patterns. A skewed XCI could be an important factor, as it regulates the expression of the mutated *MECP2* allele within the brain.^18^ In the patient described in this study, XCI studies have shown a random pattern; therefore, the phenotypic outcome cannot be explained within this context.

Besides the percentage of mosaicism, the varying levels of mutated cells in different tissues may be also contributing to a more severe than expected phenotype in the patient. For example, higher levels of cells with *MECP2* mutation may be present in the brain, as compared to blood. As brain is not an easily accessible tissue, a buccal swab would be a feasible alternative in order to get a closer estimation of the possible level of mosaicism in the brain. The utility of this approach has been demonstrated in testing for various psychiatric disorders using expression and XCI studies which have shown correlating results in buccal and brain tissue cells.^19^, ^20^ Despite the high potential value of buccal swab analysis, we were unable to obtain a sample, as the family was not available for further testing.

The introduction of Next‐generation sequencing (NGS) into routine genetic diagnosis is expected to play a key role in the future investigation of mosaicism in general. Out of all the techniques applied in this study, NGS was the only one that offered the necessary sensitivity to allow an estimation of the exact percentage of the mosaicism level, even though the presence of mosaicism was also evident by Sanger sequencing. Moreover, recent studies apply NGS to screen various patient groups including patients with RS phenotypes, revealing new mutations within *MECP2* thus proposing new pathogenic pathways.^21^ Based on the above, NGS will not only add to the list of pathogenic mutations, but also provide more data concerning the frequency of somatic mosaicism in patients with various syndromes.

The detection and thorough investigation or somatic mosaicism in RS patients as well as in other syndromes is a valuable tool for uncovering unknown mechanisms of disease development and severity assessment based on tissue specificity.

## CONFLICT OF INTEREST

The authors declare no conflict of interest.

## AUTHOR CONTRIBUTION

AA, IP, and IMA: performed DNA extraction, array‐CGH, MLPA, Sanger sequencing, restriction enzyme analysis, NGS, and XCI experiments. AT: performed bioinformatics analysis of NGS data. PE: supervised and coordinated the experimental procedures described in the manuscript. LK: collected data, reviewed literature, and drafted the manuscript. GT and VC‐A: performed clinical assessment of the patient. CS: obtained funding, conceptualized the project, and coordinated manuscript preparation and submission process. All authors contributed to manuscript writing and approved the final version of the manuscript.

## References

[1] Rett A . On a unusual brain atrophy syndrome in hyperammonemia in childhood. Wien Med Wochenschr. 1966;116(37):723‐726. 5300597

[2] Christodoulou J , Ho G . MECP2‐related disorders In: Adam MP, Ardinger HH, Pagon RA, Wallace SE, Bean LJH, Stephens K, Amemiya A, eds. GeneReviews((R)). Seattle, WA: University of Seattle; 1993:1993‐2018.

[3] Diagnostic criteria for Rett syndrome. The Rett Syndrome Diagnostic Criteria Work Group. Ann Neurol. 1988;23(4):425‐428. 2454607 10.1002/ana.410230432

[4] Neul JL , Kaufmann WE , Glaze DG , et al. Rett syndrome: revised diagnostic criteria and nomenclature. Ann Neurol. 2010;68(6):944‐950. 21154482 10.1002/ana.22124 PMC3058521

[5] Amir RE , Van den Veyver IB , Wan M , Tran CQ , Francke U , Zoghbi HY . Rett syndrome is caused by mutations in X‐linked MECP2, encoding methyl‐CpG‐binding protein 2. Nat Genet. 1999;23(2):185‐188. 10508514 10.1038/13810

[6] Evans JC , Archer HL , Colley JP , et al. Early onset seizures and Rett‐like features associated with mutations in CDKL5. Eur J Hum Genet. 2005;13(10):1113‐1120. 16015284 10.1038/sj.ejhg.5201451

[7] Mencarelli MA , Spanhol‐Rosseto A , Artuso R , et al. Novel FOXG1 mutations associated with the congenital variant of Rett syndrome. J Med Genet. 2010;47(1):49‐53. 19578037 10.1136/jmg.2009.067884

[8] Olson HE , Tambunan D , LaCoursiere C , et al. Mutations in epilepsy and intellectual disability genes in patients with features of Rett syndrome. Am J Med Genet A. 2015;167A(9):2017‐2025. 25914188 10.1002/ajmg.a.37132 PMC5722031

[9] Percy AK , Lane J , Annese F , Warren H , Skinner SA , Neul JL . When Rett syndrome is due to genes other than MECP2. Transl Sci Rare Dis. 2018;3(1):49‐53. 29682453 10.3233/TRD-180021 PMC5900556

[10] Villard L . MECP2 mutations in males. J Med Genet. 2007;44(7):417‐423. 17351020 10.1136/jmg.2007.049452 PMC2597995

[11] Tokaji N , Ito H , Kohmoto T , et al. A rare male patient with classic Rett syndrome caused by MeCP2_e1 mutation. Am J Med Genet A. 2018;176(3):699‐702. 29341476 10.1002/ajmg.a.38595

[12] Topcu M , Akyerli C , Sayi A , et al. Somatic mosaicism for a MECP2 mutation associated with classic Rett syndrome in a boy. Eur J Hum Genet. 2002;10(1):77‐81. 11896459 10.1038/sj.ejhg.5200745

[13] Bianciardi L , Fichera M , Failla P , et al. MECP2 missense mutations outside the canonical MBD and TRD domains in males with intellectual disability. J Hum Genet. 2016;61(2):95‐101. 26490184 10.1038/jhg.2015.118 PMC4770571

[14] Pieras JI , Munoz‐Cabello B , Borrego S , et al. Somatic mosaicism for Y120X mutation in the MECP2 gene causes atypical Rett syndrome in a male. Brain Dev. 2011;33(7):608‐611. 20970936 10.1016/j.braindev.2010.09.012

[15] Allen RC , Zoghbi HY , Moseley AB , Rosenblatt HM , Belmont JW . Methylation of HpaII and HhaI sites near the polymorphic CAG repeat in the human androgen‐receptor gene correlates with X chromosome inactivation. Am J Hum Genet. 1992;51(6):1229‐1239. 1281384 PMC1682906

[16] Charman T , Neilson TC , Mash V , et al. Dimensional phenotypic analysis and functional categorisation of mutations reveal novel genotype‐phenotype associations in Rett syndrome. Eur J Hum Genet. 2005;13(10):1121‐1130. 16077736 10.1038/sj.ejhg.5201471

[17] Cheadle JP , Gill H , Fleming N , et al. Long‐read sequence analysis of the MECP2 gene in Rett syndrome patients: correlation of disease severity with mutation type and location. Hum Mol Genet. 2000;9(7):1119‐1129. 10767337 10.1093/hmg/9.7.1119

[18] Braunschweig D , Simcox T , Samaco RC , LaSalle JM . X‐Chromosome inactivation ratios affect wild‐type MeCP2 expression within mosaic Rett syndrome and Mecp2‐/+ mouse brain. Hum Mol Genet. 2004;13(12):1275‐1286. 15115765 10.1093/hmg/ddh142

[19] de Hoon B , Monkhorst K , Riegman P , Laven JS , Gribnau J . Buccal swab as a reliable predictor for X inactivation ratio in inaccessible tissues. J Med Genet. 2015;52(11):784‐790. 26220467 10.1136/jmedgenet-2015-103194 PMC4680131

[20] Smith AK , Kilaru V , Klengel T , et al. DNA extracted from saliva for methylation studies of psychiatric traits: evidence tissue specificity and relatedness to brain. Am J Med Genet B Neuropsychiatr Genet. 2015;168B(1):36‐44. 25355443 10.1002/ajmg.b.32278 PMC4610814

[21] Vidal S , Brandi N , Pacheco P , et al. The utility of Next Generation Sequencing for molecular diagnostics in Rett syndrome. Sci Rep. 2017;7(1):12288. 28947817 10.1038/s41598-017-11620-3 PMC5613000

