# Supplementary material for: De novo mosaic MECP2 mutation in a female with Rett syndrome
Source: Clin Case Rep. 2019 Jan 15;7(2):366–70. doi: 10.1002/ccr3.1985 (PMC6389470; doi:10.1002/ccr3.1985)
Supplement: Supplementary file 1 [file CCR3-7-366-s001.docx]

**Appendix S1. Supplementary data**

**Genetic testing**

Mutation analysis of all four coding exons and intron/exon boundaries of the *MECP2* gene were carried out using PCR amplification and direct sequencing in six fragments: primer sequences were extracted from the study of Cheadle *et al*. 2000 ([Cheadle et al., 2000](#_ENREF_1)) and additional primer sequences were designed using primer Primer3 web interface tool (<http://bioinfo.ut.ee/primer3-0.4.0/>) and synthesized by Metabion, Planegg, Germany. Primer sequences are presented in Supplementary table I.

PCR amplifications were carried-out using standard protocols and visualized by agarose gel electrophoresis.

PCR products were purified using ExoSAP-IT^®^ (Affymetrix, Santa Clara, CA, USA) and then cycle sequenced using Big Dye Terminator Cycle Sequencing kit V3.1. Cycle sequencing cleanup was performed using Performa® DTR Gel Filtration Cartridges (EdgeBio, Gaithersburg, MD, USA), reactions were run on a 3130xl Genetic Analyzer (Applied Biosystems) and analysed with Sequencing analysis V.5.4 software (Applied Biosystems). Sequences in FASTA format were aligned to the reference genome using BLAT tool from the UCSC Genome Browser (<https://genome.ucsc.edu/cgi-bin/hgBlat>) ([Kent, 2002](#_ENREF_2)), for exon 2-4 were also aligned to NCBI Reference Sequence; NM_004992.3 using the Seqscape V2.7 analysis software (Applied Biosystems).

**Supplementary table I:** Primer sequences used for mutation analysis of all four coding exons and intron/exon boundaries of the *MECP2* gene.

| Exon | Primer number | Forward primer sequence 5’→3’ | Reverse primer sequence 5’→3’ |
| --- | --- | --- | --- |
| 1 | 1 | GGGGGAGGGTAGAGAGGAG | CCATCACAGCCAATGACG |
| 2 | 2 | GGCACAGTTTGGCACAGTTA | AAATAGCCCTGGGAAAAAGG |
| 3 | 3 | TGCCTCTGCTCACTTGTTCT | TGCCCTGTAGAGATAGGAGTT |
| 4 | 4 | AGCGTCTGCAAAGAGGAGAA | ACTTCTGGCCCTGGTTAGGT |
| 4 | 5 | TCCACCCAGGTCATGGTGATC | TCCACAGGCTCCTCTCTGTT |
| 4 | 6 | ATCCGCTCTGCCCTATCTCT | GGGCTTCACCACTTCCTTGAC |

**NGS bioinformatics analysis**

Initial pre-processing of reads was performed by the wrapper Trim_Galore tool (<https://www.bioinformatics.babraham.ac.uk/projects/trim_galore/>; version 0.3.7) which uses FASTQC ((<https://www.bioinformatics.babraham.ac.uk/projects/fastqc/>; version 0.10.1) for applying quality and cutadapt([Marcel, 2011](#_ENREF_4)); version 1.5) for trimming adapters and low quality bases. The BWA algorithm (<http://bio-bwa.sourceforge.net/>) and specifically BWA-mem (version 0.7.4) was used for sequence alignment, in order to map the reads to the GRCh37/hg19 human reference genome. Alignment post-processing was performed with Samtools ([Li & Durbin, 2009](#_ENREF_3)) (version 0.1.18), Picard package of tools (<https://broadinstitute.github.io/picard/> ; version 1.109) and GATK tools (version 3.3). In more detail, optical and PCR duplicates were marked with Picard MarkDuplicates tool; recalibration was performed by GATK BaseRecalibrattor and variant calling was done by GATK HaplotypeCaller. Further filtering was carried out using GATK Hardfiltering tool with the following parameters for SNPs: "QD < 2.0 , FS > 60.0 , MQ < 40.0 , MQRankSum < -12.5 , ReadPosRankSum < -8.0” , and for indels: "QD < 2.0 , FS > 200.0, ReadPosRankSum < -20.0. For annotation and prioritization filtering, variant effect predictor (VEP) ([McLaren et al., 2010](#_ENREF_5)) (version 81) and GEMINI ([Paila, Chapman, Kirchner, & Quinlan, 2013](#_ENREF_6)) (version 0.20.0) were used. The reads (BAM files) and percentage of mosaicism could be visualized in IGV ([Robinson et al., 2011](#_ENREF_7)) (version 2.3.40).

**References**

Cheadle, J. P., Gill, H., Fleming, N., Maynard, J., Kerr, A., Leonard, H., . . . Clarke, A. (2000). Long-read sequence analysis of the MECP2 gene in Rett syndrome patients: correlation of disease severity with mutation type and location. *Hum Mol Genet, 9*(7), 1119-1129.

Kent, W. J. (2002). BLAT--the BLAST-like alignment tool. *Genome Res, 12*(4), 656-664. doi: 10.1101/gr.229202

Li, H., & Durbin, R. (2009). Fast and accurate short read alignment with Burrows-Wheeler transform. *Bioinformatics, 25*(14), 1754-1760. doi: 10.1093/bioinformatics/btp324

Marcel, M. (2011). Cutadapt removes adapter sequences from high-throughput sequencing reads. *EMBnet.journal, 17*(1).

McLaren, W., Pritchard, B., Rios, D., Chen, Y., Flicek, P., & Cunningham, F. (2010). Deriving the consequences of genomic variants with the Ensembl API and SNP Effect Predictor. *Bioinformatics, 26*(16), 2069-2070. doi: 10.1093/bioinformatics/btq330

Paila, U., Chapman, B. A., Kirchner, R., & Quinlan, A. R. (2013). GEMINI: integrative exploration of genetic variation and genome annotations. *PLoS Comput Biol, 9*(7), e1003153. doi: 10.1371/journal.pcbi.1003153

Robinson, J. T., Thorvaldsdottir, H., Winckler, W., Guttman, M., Lander, E. S., Getz, G., & Mesirov, J. P. (2011). Integrative genomics viewer. *Nat Biotechnol, 29*(1), 24-26. doi: 10.1038/nbt.1754
